# *GISToscopy*

**DOI:** 10.1002/ccr3.1695

**Published:** 2018-07-23

**Authors:** Vincent Zimmer, Frank Lammert

**Affiliations:** ^1^ Department of Medicine II Saarland University Medical Center Saarland University Homburg Germany

**Keywords:** esophago‐gastro‐duodenoscopy, gastrointestinal stromal tumor, upper gastrointestinal bleeding

## Abstract

Gastrointestinal stromal tumors (GIST) tumors are a rare, though typical cause for upper GI bleeding. Excavated ulcerated lesions are common; however, a clear‐cut visualization of the tumor surface (“*GISToscopy*”) itself has not yet been reported. In line, careful biopsy taking streamlines the diagnostic work‐up by providing pathologic diagnosis.

An 82‐year‐old woman presented with signs of acute upper GI bleeding. The patient underwent urgent upper endoscopy, revealing a doughnut‐shaped deeply excavated protruding lesion in the gastric antrum just below the incisura angularis with minor stigmata of recent hemorrhage (Figure 1A,B). Further scrutinization of the lesion with close‐up views into the cavity indicated a glazed whitish surface with visible irregular capillaries, reminiscent of the highly vascular nature of gastrointestinal stromal tumors (GIST) (Figure 1C,D). Conventional endoscopic biopsies were taken from the center and the periphery, and biopsy sites were prophylactically injected with a diluted suprarenin mixture. No bleeding complications occurred during further follow‐up. Histopathology confirmed the presence of a spindle cell‐type, low‐mitotic GIST tumor with CD34 and CD117 positivity. A CT scan showed the gastric wall tumor measuring 5 cm and excluded distant spread; hence, the patient was referred for laparoscopic tumor resection.

**Figure 1:**
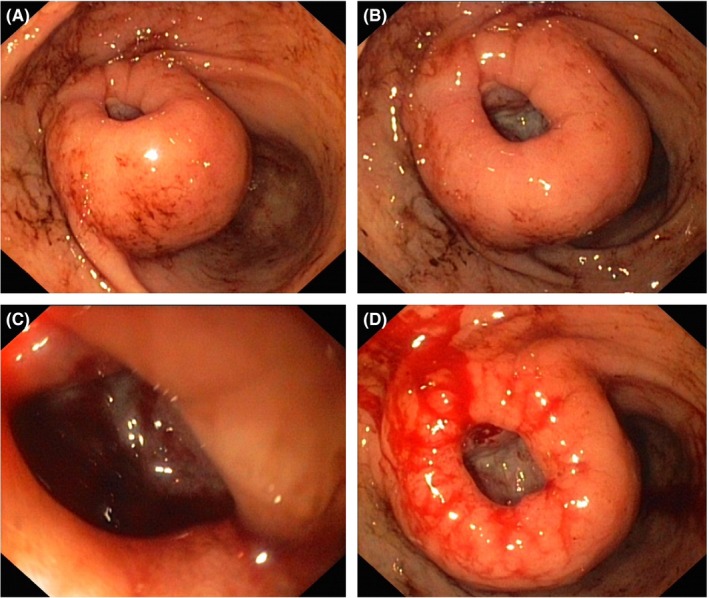
Endoscopic view of the distal stomach revealing (A) an excavating protruding lesion with (B) a doughnut‐like appearance. (C) Close‐up and (D) frontal view with direct visualization of the tumor surface exhibiting a glazed whitish surface with aberrant tumor capillaries

Notwithstanding that endoscopic GIST presentations are myriad, clear‐cut endoscopic visualization of the tumor surface itself, most likely resulting from extensive pressure‐induced mucosal necrosis, is uncommon and may turn a routine gastroscopy into an insightful and diagnostically straightforward “*GISToscopy,*” which is in contrast to the more frequently observed fibrin‐covered ulcerated endoscopic GIST appearances.

## CONFLICT OF INTEREST

None declared.

## AUTHORSHIP

VZ: involved in clinical and endoscopic management and drafted and finalized the manuscript. FL: involved in clinical management, revision, and finalization of the manuscript.

